# Habituation of the C-Start Response in Larval Zebrafish Exhibits Several Distinct Phases and Sensitivity to NMDA Receptor Blockade

**DOI:** 10.1371/journal.pone.0029132

**Published:** 2011-12-28

**Authors:** Adam C. Roberts, Jun Reichl, Monica Y. Song, Amanda D. Dearinger, Naseem Moridzadeh, Elaine D. Lu, Kaycey Pearce, Joseph Esdin, David L. Glanzman

**Affiliations:** 1 Department of Integrative Biology and Physiology, University of California Los Angeles, Los Angeles, California, United States of America; 2 Undergraduate Interdepartmental Neuroscience Program, University of California Los Angeles, Los Angeles, California, United States of America; 3 Department of Molecular, Cell, and Developmental Biology, University of California Los Angeles, Los Angeles, California, United States of America; 4 Department of Neurobiology and the Brain Research Institute, David Geffen School of Medicine at University of Calfornia Los Angeles, Los Angeles, California, United States of America; Max-Planck-Institut für Neurobiologie, Germany

## Abstract

The zebrafish larva has been a valuable model system for genetic and molecular studies of development. More recently, biologists have begun to exploit the surprisingly rich behavioral repertoire of zebrafish larvae to investigate behavior. One prominent behavior exhibited by zebrafish early in development is a rapid escape reflex (the C-start). This reflex is mediated by a relatively simple neural circuit, and is therefore an attractive model behavior for neurobiological investigations of simple forms of learning and memory. Here, we describe two forms of short-lived habituation of the C-start in response to brief pulses of auditory stimuli. A rapid form, persisting for ≥1 min but <15 min, was induced by 120 pulses delivered at 0.5–2.0 Hz. A more extended form (termed “short-term habituation” here), which persisted for ≥25 min but <1 h, was induced by spaced training. The spaced training consisted of 10 blocks of auditory pulses delivered at 1 Hz (5 min interblock interval, 900 pulses per block). We found that these two temporally distinguishable forms of habituation are mediated by different cellular mechanisms. The short-term form depends on activation of *N*-methyl-d-aspartate receptors (NMDARs), whereas the rapid form does not.

## Introduction

A major goal of modern neuroscience is to characterize the physical changes within the nervous system that underlie learning and memory. Significant progress has been made in mammalian systems toward identifying potential neuronal substrates of memory [1]–[4], and molecular techniques are now available for labeling specific neurons that participate in the memory engram for some types of learning [5], [6]. Despite these advances, cataloging all of the cellular and molecular processes that mediate sophisticated forms of learning in the enormously complex mammalian brain is, at present, a quixotic enterprise. To more readily achieve the goal of linking neuronal modifications to learned behavioral changes, we have chosen to study elementary learning in an inframammalian vertebrate, the zebrafish.

The zebrafish has several attributes that make it particularly attractive as a model organism for biological investigations of behavior. Among these are rapid development, high fecundity, and ease of genetic manipulation [7], [8]. Another significant advantage of the zebrafish is that it is transparent in the larval stage, making it ideally suited for optical and optogenetic investigations of neuronal function [9]–[12]. Finally, although a vertebrate with complex vertebrate behavior [13], zebrafish exhibit some simple behaviors that are regulated by relatively simple neural circuits, circuits that are highly amenable to neurophysiological analyses [14], [15]. One such behavior is the startle response. This rapid escape response (the C-start) is mediated by a well-defined neural circuit in the brainstem and spinal cord; a major component of this circuit is a small number of hindbrain neurons, the most prominent of which are the large, bilaterally paired Mauthner (M) cells [7], [16]–[19]. In adult goldfish, a close relative of the zebrafish, the C-start circuit is highly plastic [20]–[24].

In the present study we examined habituation of the C-start in the larval zebrafish. Habituation is a nonassociative form of learning during which an organism decreases its responsiveness to a repeated stimulus [25], [26]. An evolutionarily ancient form of learning, habituation is present in organisms ranging from *Cnidarians* [27] to humans [28]. But despite its simplicity and apparent ubiquity, at present we possess only a rudimentary understanding of the neurobiology of habituation [29], [30].

Short-term habituation of the C-start in zebrafish larvae was first described by Eaton and colleagues in 1977 [31]; during the intervening decades, however, there has been no in-depth investigation of this form of learning. A recent study by Best and colleagues [32] examined habituation of escape-related movements by larval zebrafish in response to auditory stimuli. But these investigators did not use high-speed videography to record the movement of the fish. This is mechanistically problematic because zebrafish can generate an escape response through non-M-cell neural circuits [18], [33], [34]; unless one makes direct electrophysiological or optical recordings of the M-cell's action potential, the only reliable method for distinguishing between the M-cell-mediated and non-M-cell-mediated escape responses is latency of response onset: the M-cell mediated escape (the C-start) has a significantly shorter onset latency (<12 ms) than does the non-M-cell-mediated response (mean ∼28 ms) [33], [34] (but see Ref. [18]). Best and colleagues did not attempt to distinguish between the short-latency and long-latency escapes in their behavioral study, and therefore could not know whether or not the responses of the animals were the consequence of M-cell firing.

We have performed a comprehensive parametric study of short-lasting habituation of the C-start in zebrafish larvae, and have begun to analyze its underlying mechanisms. Here we present evidence that there are distinct forms of short-lasting habituation of the C-start in the larvae. In addition, we demonstrate a critical role for *N*-methyl-d-aspartate receptors (NMDARs) [3], [35], [36] in one of these forms.

## Methods

### Ethics Statement

All experimental procedures in this study were approved by the Chancellor's Animal Research Committee (ARC) at the University of California at Los Angeles (#2005-053-21).

### Animals and behavioral apparatus

**Figure 1:**
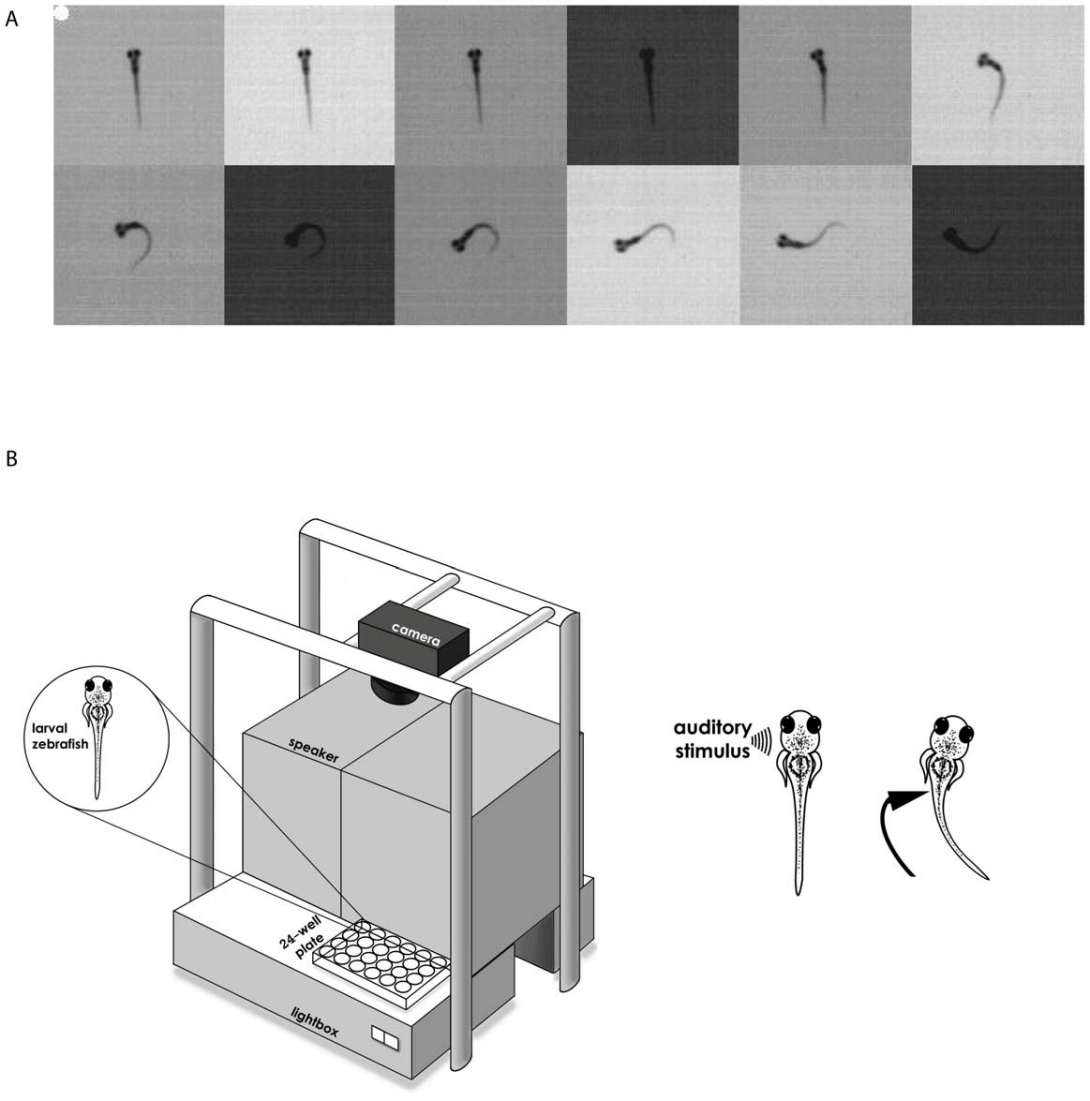
The C-start reflex in larval zebrafish and the experimental apparatus used to study habituation of the reflex. (**A**) Representative C-start reflex in a zebrafish larva. Frames commence at the presentation of an auditory/vibrational (AV) pulse; the white dot indicates the initiation of the C-start reflex. Images were recorded at 1000 frames/s. For illustration purposes, only every other frame is shown. (**B**) Experimental apparatus used to elicit the C-start. The plastic wells containing the zebrafish were placed on top of a light box, which was used to achieve adequate visual contrast during video recording. Each well was filled with ∼3 ml of E3 and contained one zebrafish. We were able to record the responses of as many as 24 zebrafish in a single session.

Zebrafish eggs were collected after standard breeding protocols, and placed into E3 solution (5 mM NaCl, 0.33 mM MgCl_2_, 0.33 mM CaCl_2_, 0.17 mM KCl, 10^−5^% methylene blue, pH 7.2) in an incubator (28.5°) to allow the embryos to develop. Wild-type TL zebrafish, obtained from the core facility at the University of California, Los Angeles (UCLA), were used for all experiments. To image the zebrafish escape response, we used one of two high-speed cameras. For the experiments investigating pharmacological manipulation of rapid habituation (below), a Casio Exilim ExFH25 (Casio America, Dover, NJ) was used, and images were recorded at 240 frames/s. For all other experiments, we employed the TroubleShooter TS100MS (Fastec Imaging, San Diego, CA), and recorded at 1000 frames/s. In order to achieve sufficient visual contrast to detect the escape responses (Fig. 1A) in the high-speed recordings of their behavior, the larvae had to be illuminated with intense light. Therefore, the larvae were put into individual wells, each containing 3 ml of E3 solution, and the wells were placed on a light box (Gagne Inc., Johnson City NY). A speaker, positioned next to the wells, was used to deliver auditory/vibrational (AV) stimulation (Fig. 1B). The fish were permitted to acclimate to the wells for 1 h prior to the start of the experiments. 1 ms-long (200 Hz ramp wave), 109 dB auditory pulses, produced with a function generator (NS-R2001; Leader, Cypress, CA) and amplifier (Insignia, Richfield, MN), were used to elicit startle responses. A response to an auditory pulse was scored as a startle response when the animal began the characteristic C-bend within 25 ms after the stimulus.

### Behavioral protocols and statistical analyses

In the experiments involving drug treatment, the fish were housed in individual wells with drug or control solutions for 24 h prior to the start of the experiment. Each fish was given paramecia for the first 23 h of this period, after which the fresh experimental solutions were introduced into the wells. In the experiments with dl-2-amino-5-phosphonopentanoic acid (APV), the drug was dissolved directly into E3. Animals were maintained in drug-containing or control wells throughout the experiment. APV was obtained from Sigma (St. Louis, MO).

- Rapid habituation. After a 1-h acclimation period, zebrafish were given habituation training (120 pulses at 1 Hz, 109 dB). In one set of experiments C-starts were measured throughout the training period. In a second set of experiments the same training protocol was used, but habituation memory was tested 10 s, 1 min, and (in some experiments) 15 min after training. To assess the significance of group differences, the number of responses that occurred during the training period, or on the posttest, were compared using a two-tailed, unpaired *t*-test.
- Short-term habituation. A more persistent form of habituation was elicited by giving larvae spaced training. After a 1-h acclimation period, three pretests (5 min ISI, 109 dB) were performed. Five min after the final pretest, the larvae were given extended habituation training, which consisted of 10 blocks (900 pulses, 1 Hz) of spaced (5 min interblock interval) AV stimulation. Fifteen min after the last block of stimuli, the zebrafish received three posttests (5 min ISI, 109 dB). To determine the amount of habituation produced by the training, we calculated a habituation index (HI). The HI was the posttest response rate (average of the three posttests) minus the pretest response rate (average of the three pretests). Two-tailed, unpaired *t*-tests, one-way ANOVAs, and repeated measures ANOVAs were used for the statistical comparisons. Student Newman Keuls tests were used for post-hoc analyses when the results of the ANOVA were significant.

## Results

**Figure 2:**
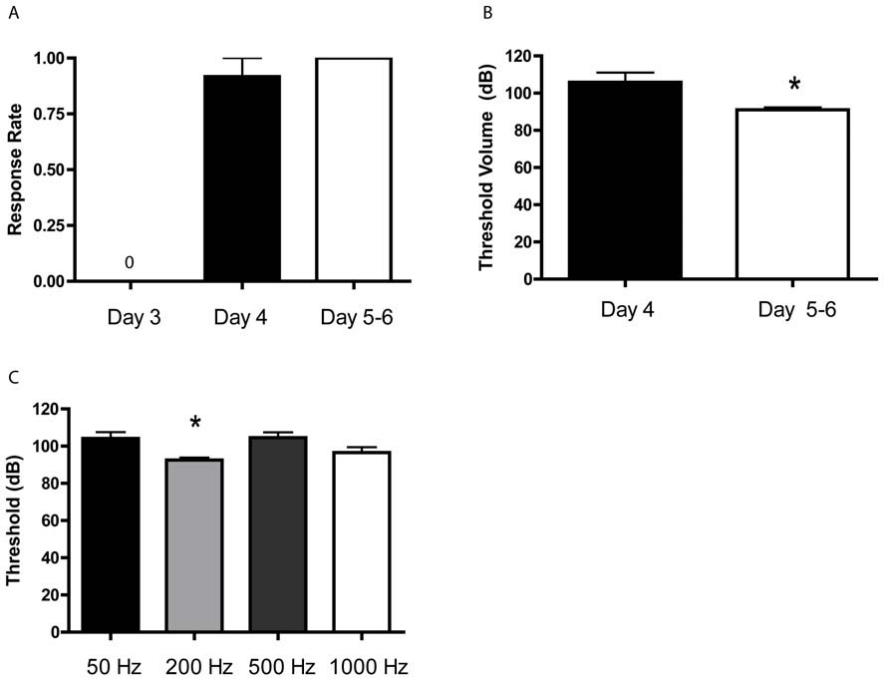
Zebrafish do not exhibit a C-start response to auditory stimuli before 4 dpf, and subsequently are most responsive to a 200-Hz auditory stimulus. (**A**) Mean response rate of larval zebrafish to AV stimulation during development. No escape responses were elicited 3 dpf (*n* = 8), whereas the response rate at 4 dpf and 5–6 dpf was 0.92 and 1.0, respectively. (**B**) Mean volume (dB) of auditory pulses that elicit a C-start response in larvae 4 dpf (*n* = 12) and 5–6 dpf (*n* = 8). It required significantly stronger auditory stimulation to evoke a C-start in the younger fish than in the older fish (*t*_[18]_ = 2.28, *p*<0.05). One 4 dpf zebrafish failed to respond to auditory pulses, and therefore were assigned a threshold of 135 dB, the highest volume tested. (Data in Figures 2A and 2B are from the same experiments.) The asterisk indicates a significant difference between the groups. Error bars in this and subsequent graphs represent SEM. (**C**) Mean volume (dB) of sound pulses of different auditory frequencies used to elicit a C-start reflex: 50 Hz (*n* = 8), 200 Hz (*n* = 8), 500 Hz (*n* = 8), and 1000 Hz (*n* = 8). A one-way ANOVA performed on the group data was significant (*F*_[3,28]_ = 4.58, *p*<0.01). The mean threshold to elicit escape responses was 104.13±3.38 dB in the 50 Hz group, 92.5±1.32 dB in the 200 Hz group, 104.5±2.93 dB in the 500 Hz group, and 96.5±2.93 dB in the 1000 Hz group. SNK post hoc tests showed that a 200 Hz stimulus elicited escape responses at a significantly lower threshold than did either the 50 Hz stimulus or the 500 Hz stimulus (*p*<0.05), as indicated by the asterisk.

Initially, we determined the developmental age at which larval zebrafish were able to perform an escape response to AV stimuli. Although morphological contacts between the primary auditory nerve and the M-cell form early in development [37], [38], and sound-evoked postsynaptic currents are observed as early as 40 h post-fertilization [38], we failed to observe escape responses at 3 d post-fertilization (dpf) (Fig. 2A). We first elicited escape responses at 4 dpf, but the auditory threshold at which these responses were elicited was significantly higher than that for older fish (5–6 dpf, *p*<0.05), and the responses were more variable (Fig. 2B). The mean threshold (dB) for eliciting an escape was 105.83±5.19 dB in 4 dpf larval zebrafish and 91±1.31 dB in 5–6 dpf zebrafish. Accordingly, we restricted our experiments to larvae ages 6–8 dpf. Similar to the results of a previous study that investigated escape behavior in zebrafish larvae [32], we found that the best stimulus frequency for eliciting an escape response was 200 Hz (Fig. 2C) (one-way ANOVA, *p*<0.05), although escape responses could be elicited by a wide range of frequencies (50–1000 Hz).

### Characterization of rapid habituation

**Figure 3:**
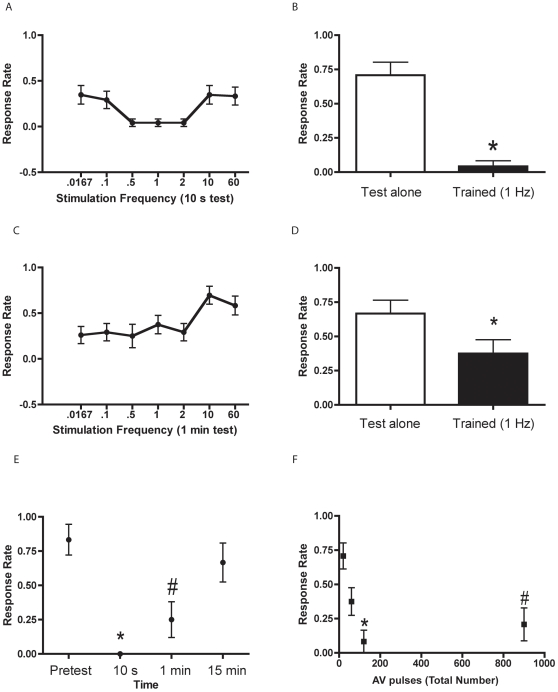
Low-frequency stimulation is more effective at eliciting rapid habituation than high-frequency stimulation. (**A**) Mean response rates of larval zebrafish (6–7 dpf) after 120 pulses of AV stimulation at different frequencies, tested 10 s after the last training pulse. The response rates were: 0.0167 Hz, 0.35±0.10 (*n* = 23); 0.1 Hz, 0.29±0.10 (*n* = 24); 0.5 Hz, 0.04±0.04 (*n* = 24); 1 Hz, 0.04±0.04 (*n* = 24); 2 Hz, 0.04±0.04 (*n* = 24); 10 Hz, 0.35±0.10 (*n* = 23); and 60 Hz, 0.33±0.10, (*n* = 24). (**B**) Mean rate of C-start responses 10 s after habituation training (1 Hz, 120 pulses). The Trained group (*n* = 24) exhibited a significantly lower response rate than did the Test alone (*n* = 24; *t*_[46]_ = 6.44, *p*<0.0001), as indicated by the asterisk. The data for the Trained group came from the experiments shown in (A). (**C**) Mean response rates of larval zebrafish to pulses of different auditory frequencies, tested 1 min after training. The data are from the same experiments presented in (A). The response rates were: 0.0167 Hz, 0.26±0.09 (*n* = 23); 0.1 Hz, 0.29±0.10 (*n* = 24); 0.5 Hz, 0.25±0.13 (*n* = 24); 1 Hz, 0.38±0.10 (*n* = 24); 2 Hz, 0.29±0.10 (*n* = 24); 10 Hz, 0.70±0.10 (*n* = 23); and 60 Hz, 0.58±0.10 (*n* = 24). (**D**) Mean response rate 1 min after habituation training (1 Hz, 120 pulses). The Trained group (*n* = 24) exhibited significantly greater habituation than did the Test alone group (*n* = 24; *t*_[46]_ = 2.07, *p*<0.05), as indicated by the asterisk. The data for the Trained group were from the experiments in (C). (**E**) Persistence of rapid habituation following training at 1 Hz (120 pulses). The pretest response rate was based on the response to the first stimulus of the habituation training, and posttest rates were based on the responses of the same animals (*n* = 12) at 10 s, 1 min, and 15 min after training. To determine when the response rate returned to baseline levels, we performed a repeated measures ANOVA (*F*_[3,33]_ = 12.72, *p*<0.0001). SNK post hoc tests used to probe for significant differences among the responses to the various tests indicated that habituation was present at 10 s and 1 min after training compared to the pretest and 15 min posttest (*p*<0.05 for each comparison), whereas no habituation was present at the 15 min posttest compared to the pretest response rate (*p*>0.05). The asterisk indicates a significant difference between the 10 s test and the pretest and 15 min test values, whereas a pound sign indicates a significant difference between the 1 min test and the pretest and 15 min test values. (**F**) Number of pulses required to elicit rapid habituation (1-Hz stimulation). To determine the most effective number of pulses to elicit habituation (1-min posttest), we first performed a one-way ANOVA, which showed that the groups differences were significant (*F*_[3, 92]_ = 9.89, *p*<0.0001). SNK post hoc tests indicated that the group that received 120 AV pulses had a significantly lower response rate than the groups that received only 20 or 60 AV pulses (*p*<0.05 for each comparison), as indicated by an asterisk. The group that received 900 pulses was significantly less responsive than the group that received 20 pulses (*p*<0.05), as indicated by a pound sign.

To determine the experimental protocol that most effectively elicited rapid habituation, we stimulated larval zebrafish with 120 auditory pulses at different frequencies and tested the animals' responsiveness at 10 s and 1 min after the last pulse. AV stimuli at frequencies ranging from 0.5–2 Hz were most effective in habituating the escape response, as assayed 10 s after training (Fig. 3A). Interestingly, lower (0.0167–0.1 Hz) and higher (10–60 Hz) stimulation frequencies produced less habituation. We also included a control group (Test alone group) that received the same testing protocol as the Trained group, but was not given habituation training. We compared a Test alone group (mean response rate of 71.0±0.10) to a Trained group (mean response rate of 0.04±0.04) that received 1-Hz habituation training. The difference between the two groups was highly significant on the 10-s posttest (*p*<0.0001, Fig. 3B). We found that a relatively broad range of stimulus frequencies could produce habituation at 1 min after training. In fact, as shown in Figure 3C, all low-frequency stimulation protocols (0.0167–2 Hz) yielded similar amounts of habituation; the higher frequency stimulation protocols, however, were less effective at inducing habituation. The mean response rate on the 1-min posttest was 0.38±0.10 in the Trained group and 0.67±0.10 in the Test alone group. A planned comparison between these two groups for the 1-min posttest indicated that the training produced significant habituation (*p*<0.05, Fig. 3D).

We next sought to determine the time course of the habituation memory. We measured the escape response at 10 s, 1 min and 15 min after habituation training (120 pulses delivered at 1 Hz). We found that the C-start was habituated for at least 1 min posttraining (Fig. 3E), and afterwards returned to pretest levels (defined as the animal's responsiveness to the first pulse of habituation training) within 15 min (repeated measures ANOVA, *p*<0.05). The mean response rates were: pretest, 0.83±0.11 (*n* = 12): 10 sec, 0.00±0.00 (*n* = 12); 1 min, 0.25±0.13 (*n* = 12); and 15 min, 0.67±0.14 (*n* = 12). To ascertain the most effective number of auditory pulses for eliciting rapid habituation, we measured habituation at the 1-min posttest after varying the number of auditory pulses from 20–900. We found that 120 pulses of auditory stimuli were the most effective at eliciting habituation; no additional habituation was achieved with further stimulation (900 pulses) (one-way ANOVA, *p*<0.05; Fig. 3F). The mean response rates after training with varying numbers of pulses were: 20 pulses, 0.71±0.10 (*n* = 24); 60 pulses, 0.38±0.10 (*n* = 24); 120 pulses, 0.08±0.06 (*n* = 24); and 900 pulses, 0.21±0.09 (*n* = 24).

### The role of NMDAR-mediated transmission in rapid habituation

**Figure 4:**
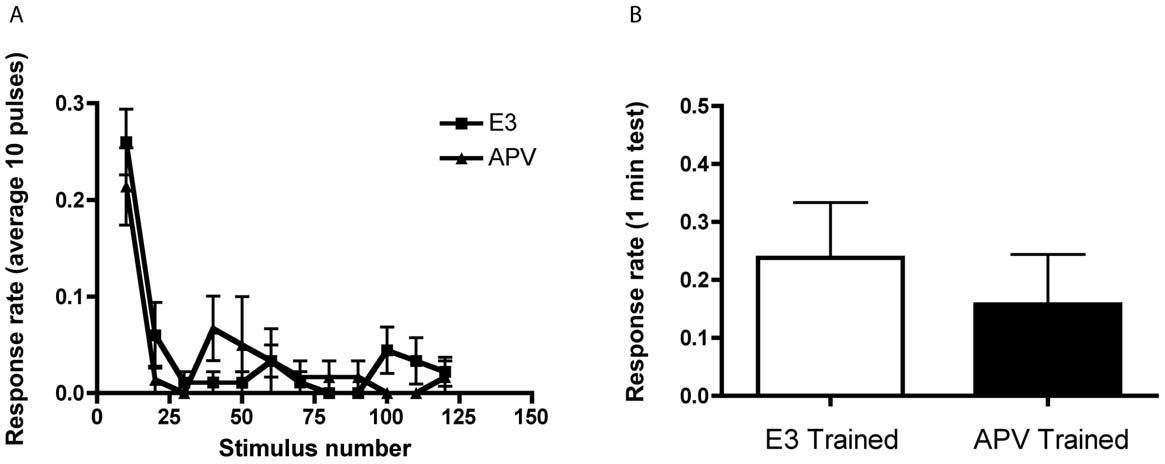
Pharmacological antagonism of NMDA receptors does not disrupt rapid habituation. (**A**) Response rates during habituation training (1 Hz, 120 pulses) in APV Trained (*n* = 7) and E3 Trained (*n* = 9) larvae. Blockade of NMDARs did not alter habituation to the first 10 AV stimuli. The response rate of the APV Trained group was 0.21±0.04, whereas that of the E3 Trained group was 0.26±0.03 (*t*_[14]_ = 0.79, *p*>0.4). (**B**) In addition, there was no difference between the APV Trained group (*n* = 19) and the E3 Trained group (*n* = 21) on the 1-min posttest (*t*_[38]_ = 0.62, *p*>0.5). (Note that the data in this graph include fish from [A], in which the responses during training were recorded and other fish in which only the posttest response was recorded.)

We exploited the ability of larval zebrafish to passively absorb drugs from their environment [39] in order to test the involvement of NMDAR-dependent activity in rapid habituation of the C-start. We incubated zebrafish larvae in the NMDAR antagonist APV (100–200 µM in E3) for 24 h; control larvae were incubated in E3 alone. The fish were then trained with 120 auditory pulses at 1 Hz. Blockade of NMDARs had no effect on the responses of the fish during training (*p*>0.05, Fig. 4A). Furthermore, a comparison of the responsiveness of APV-treated (0.16±0.09) and control animals (0.24±0.10) at 1 min posttraining also showed no significant difference between the two groups (*p*>0.5, Fig. 4B). We have confidence in this negative result because we found that the same concentration of APV did block more persistent habituation (below).

### Demonstration of short-term habituation

**Figure 5:**
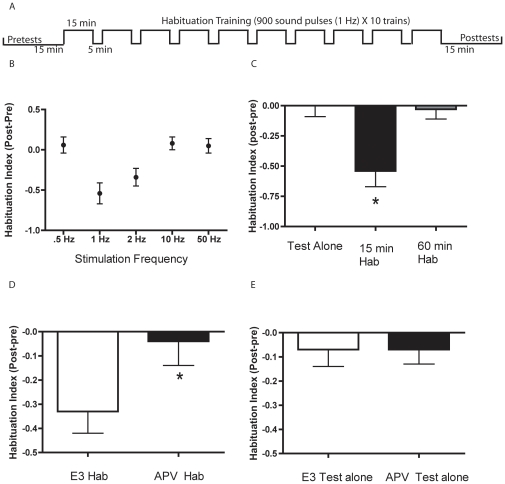
Spaced training elicited short-term habituation that depends on NMDAR activation. (**A**) Protocol for spaced training. (**B**) Habituation Indices (HI) for larvae after spaced training at different stimulus frequencies. The HI was determined by measuring the response rate of the zebrafish prior to training (3 tests, 5 min ISI) and after training (15–25 min after training; 3 tests, 5 min ISI). The posttest response rates were then subtracted from the pretest response rate to calculate the HI. The HIs for the 15–25 min posttests were: 0.5 Hz, 0.06±0.10 (*n* = 6); 1 Hz, −0.54±0.13 (*n* = 8); 2 Hz, −0.34±0.11 (*n* = 8); 10 Hz, 0.08±0.08 (*n* = 8); and 50 Hz, 0.05±.09 (*n* = 7). (**C**) Mean HI after spaced training (1 Hz) during the 15–25-min and 60–70-min posttests. There were three groups: a group that was tested 15–25 min after training (15–25 min Trained, *n* = 8), a group (Test alone, *n* = 6) that did not receive habituation training, and a group (60–70 min Trained, *n* = 24) that received habituation training and was tested 60–70 min after training. A one-way ANOVA indicated that there were significant differences among the three groups (*F*_[2,35]_ = 6.76, *p*<0.01). SNK post hoc tests indicated that the 15–25 min Trained group showed greater habituation than did the Test alone and the 60–70 min Trained groups (*p*<0.05 for each comparison), as indicated by the asterisk. The data used for the 15–25 min Trained group was from the experiments presented in (A). (**D**) Blockade of NMDARs disrupted the habituation induced by spaced training. The HI of the APV Trained group (*n* = 15) was −0.04±0.10, whereas it was −0.33±0.09 for the E3 Trained group (*n* = 15) (*t*_[28]_ = 2.16; *p*<0.05). The asterisk indicates a statistically significant difference. (E) APV treatment did not disrupt the responsiveness of untrained fish. There were no differences between the HIs of the untrained (Test alone) group treated with APV (*n* = 14) and the Test alone group treated with E3 (*n* = 14). The mean HI for the untrained APV-treated group was −0.07±0.06), and −0.07±.07 for the untrained E3-treated group (*t*_[26]_ = 0.008, *p*>0.9).

Having identified a form of habituation of the C-start that lasted≥1 min, we sought protocols that would elicit longer-lasting habituation. Accordingly, we used spaced training, which has been previously shown to be more effective in eliciting prolonged habituation than massed training [40]. We found that auditory pulses delivered at 1 Hz in 10 spaced blocks of stimuli (900 pulses per block, 5 min interblock interval; Fig. 5A) produced the greatest habituation (Fig. 5B). (Massed training, 9000 pulses at 1 Hz, failed to elicit short-term memory; data not shown.) Although training at a stimulus rate of 2 Hz also elicited significant habituation, training at a lower frequency (0.5 Hz) and higher frequencies (10 and 50 Hz) were ineffective. To confirm these results, and to measure the persistence of the memory induced by spaced training, we compared the effect of 1-Hz spaced training protocol as assessed 15–25 min after training (Trained_15–25 min posttests_ group), or 60–70 min after training (Trained_60–70 min posttests_ group), with that of the test stimulation alone (Test alone group). The Trained_15–25 min posttests_ group exhibited significantly lower responsiveness (HI = −0.54±0.13) than did either the Trained_60–70 min posttests_ group (HI = −0.03±0.08) or the Test alone group (HI = 0.00±0.09, *p*<0.05, Fig. 5C). These results indicate that short-term habituation training produced memory that lasted for ≥25 min, but <1 h.

### The role of NMDAR-mediated transmission in short-term habituation

To determine whether NMDAR-mediated activity was required for short-term habituation, larval zebrafish were incubated in either 100 µM APV or control (E3) solution for 24 h prior to the start of an experiment. Following incubation in APV, larvae that received spaced training at 1 Hz did not exhibit habituation (HI = −0.04±0.10) on the 15–25-min posttests, in contrast to the control larvae incubated in E3 alone (HI = −0.33±0.09, *p*<0.05; Fig. 5D). To confirm that exposure to APV did not affect the health or responsiveness of the zebrafish, we examined zebrafish incubated for 24 h in either APV or E3, but not given habituation training. We found no significant difference in the responsiveness of the two groups of untrained fish (*p*>0.9, Fig. 5E). Thus, NMDAR activity is required for short-term (25 min-to-1-h) habituation of the C-start.

## Discussion

The present study is a systematic investigation of habituation of the M-cell-mediated escape response in larval zebrafish, a form of learning originally demonstrated by Eaton and colleagues [31] over forty years ago. The relative simplicity of the neural circuitry that underlies the C-start, together with other significant biological advantages of the zebrafish system [7], [15], [41], [42], should significantly facilitate the analysis of the cellular and molecular modifications that mediate habitation and, potentially, other elementary forms of learning and memory. (See Ref. [43] for a related approach.) Note that, despite significant advances during the past few decades in our understanding of the biology of learning and memory, we still lack a complete mechanistic scheme for any form of learning and memory [29].

Although there is evidence of synaptic connectivity between the 8^th^ nerve auditory input and the M-cell as early as 40 hpf [37], [38], we did not observe an escape response until 4 dpf, as previously reported [44]. Also similar to the findings of Best and colleagues [32], who measured auditory-elicited escape movements, we found that a 200 Hz tone was the most effective in evoking the C-start in larval zebrafish. However, we observed C-starts to a range of auditory frequencies (50–1000 Hz).

We characterized two forms of habituation of the C-start in larval (6–8 dpf) zebrafish.

One form, a rapid form lasting between 1 min and 15 min, could be reliably elicited by low-frequency auditory stimulation. Nonetheless, we observed significant variability in the amount of habituation elicited in our experiments. At present, we do not understand the source of this variability. We were unable to block the rapid form of habituation of the C-start with the NMDAR antagonist APV, although the same dose of the antagonist was able to block the more extended form of habituation (Fig. 5D, E).

It is possible that changes in electrical transmission underlie rapid habituation of the C-start. For example, transient modifications of gap junction conductance and/or gap junction number, may reduce the strength of the 8^th^ nerve input to the M-cell—which contains a significant electrical component [45]— and thereby modify the threshold for escape. Rapid, reversible uncoupling of electrical synapses is a well-described phenomenon in the central nervous system [46]–[49], and functional uncoupling of electrical synapses has been proposed as a mechanism for behavioral switching [50], [51]. Another neuronal mechanism that could explain our results is ephaptic inhibition of the M-cell through extracellular currents created by inhibitory neurons within the axon cap [52], [53]. As shown by Weiss et al. [53], the electrical field effect generated by such currents can inhibit the generation of M-cell action potentials, and thereby regulate the threshold for the elicitation of the C-start.

Feed-forward chemical inhibition is another potential cellular mechanism for rapid habituation of the escape response. However, Weiss and colleagues [53] have reported that feed-forward chemical inhibition of the M-cell occurs several milliseconds after gap junction-mediated transmission from the primary afferents of the 8^th^ nerve in adult goldfish. If similar temporal kinetics characterize feed-forward chemical inhibition in larval zebrafish, such a delay would make it unlikely that chemical inhibition contributes to rapid habituation of the C-start. However, changes in tonic chemical inhibition could play a role.

**Figure 6:**
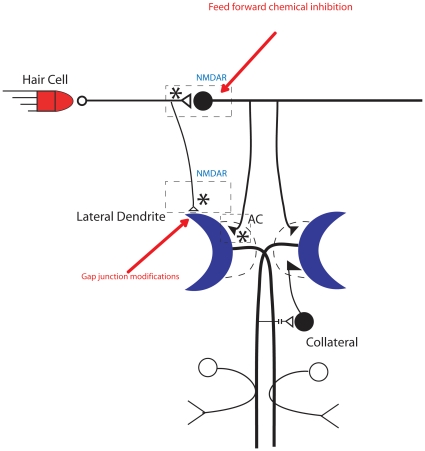
Model of the M-cell circuitry showing the potential sites of synaptic change that underlie habituation in the zebrafish. Asterisks indicate potential sites of synaptic plasticity at excitatory chemical/electrical synapses (open symbols) and inhibitory synapses (filled symbols). Nonsynaptic changes, including ephaptic inhibition of the M-cell and decreased excitability of the sensory afferents or M-cell, may also contribute to behavioral habituation. AC = axon cap.

We also demonstrated a more persistent form of habituation of the C-start response, one lasting between 25 min and 1 h. We intend in future experiments to determine, what role, if any, inhibition plays in this short-term habituation. Activity-dependent potentiation of chemical inhibitory synapses in the auditory pathway to the M-cell has been well documented [45], and has been previously linked to behavioral plasticity in fish [22]; possibly, this form of synaptic plasticity contributes to short-term habituation of the C-start. In support of this idea, we found evidence for a role for NMDARs in short-term habituation, although we do not know the synaptic location of these NMDARs. Another possibility is that habituating stimuli cause NMDAR-dependent potentiation of inhibitory electrical synapses. For example, homosynaptic potentiation of synapses made by auditory fibers of the 8^th^ nerve onto feed-forward inhibitory interneurons could increase inhibition of the M-cell, either through chemical or ephaptic inhibition (Fig. 6). (Interestingly, in the goldfish brain the NR1 subunit of the NMDAR has been reported to be present in postsynaptic densities juxtaposed to the club endings of 8^th^ nerve excitatory axons, which synapse onto the lateral dendrites of the M-cell close to gap junction plaques [55].) Finally, NMDAR-dependent [54] of the excitatory auditory inputs, either chemical or electrical (or both) [24], to the M-cell, could play a role in habituation of the C-start. Future experiments, involving calcium imaging or, possibly, electrophysiological recording, from M-cells in semi-intact zebrafish larvae, should be able to clarify the synaptic mechanisms of this simple form of learning.

In summary, the present study demonstrates that short-lasting habituation of the zebrafish C-start reflex exhibits multiple forms that are both temporally and mechanistically distinct. It also provides the basis for cellular and molecular investigations of a simple form of learning in a genetically tractable vertebrate organism. We believe that such investigations may well yield novel insights into the fundamental biological mechanisms that regulate vertebrate learning and memory.

### Addendum

While this manuscript was under review, Wolman et al. [56] published results related to ours. In particular, they characterized a form of habituation similar to our rapid habituation. Consistent with our findings, Wolman and colleagues found that 1-Hz stimulation was effective in eliciting this form of habituation. However, these investigators reported that the noncompetitive NMDAR antagonists MK-801 and ketamine enhanced the baseline response rate and blocked rapid habituation; their results contrast with those from our experiments in which we found the competitive NMDAR antagonist, APV, to be without effect on either the baseline response rate or rapid habituation (Fig. 4). To determine the source of these contrasting effects of the NMDAR antagonists, we tested the effect of MK-801 (Methods S1). We found that incubating the larvae in 100 µM MK801 for 15–45 min enhanced the baseline response rate and disrupted rapid habituation of the C-start (Fig. S1), consistent with Wolman et al.'s findings. By contrast, we replicated our original result for APV using Wolman et al.'s incubation protocol; specifically, we found that APV (≤200 µM) had no effect on the baseline response rate or on rapid habituation of the C-start (Fig. S2). There are several possible explanations for the differential effects of the NMDAR antagonists on rapid habituation. First, APV may not completely antagonize NMDARs in larval zebrafish, although notice that we did observe an effect of APV on short-term habituation of the C-start (Fig. 5). Second, the non-competitive antagonists MK-801 and ketamine may have nonspecific effects on the M-cell neurocircuitry [57]. Future experiments using genetic manipulation of NMDARs may be able to resolve the discrepancy between the results with MK-801 and ketamine, and those with APV.

## Supporting Information

Figure S1 **The noncompetitive NMDA receptor antagonist MK-801 enhances the baseline C-start response rate and disrupts rapid habituation.** (**A**) Responsiveness of zebrafish larvae after incubation with 100 µM MK-801 (*n* = 36) or E3 (*n* = 35). The response rate of the MK-801 group was 0.96±0.02, whereas that of the E3 control group was 0.85±0.04 (*t*_[69]_ = 2.60, *p*<0.05). (**B**) Responsiveness after habituation training with 30 pulses (1-Hz stimulation). The MK-801 group (*n* = 36, 0.67±0.08) was significantly more responsive than the E3 group (*n* = 35, 0.23±0.07) (*t*_[69]_ = 4.07, *p*<0.001) when tested 10 s after the last auditory pulse. (**C**) The results following training with 120 auditory pulses (also at 1 Hz) were similar to those with the 30-stimuli protocol. Again, the MK-801 group (*n* = 36, 0.75±0.07) responded at a higher rate than did the E3 group (*n* = 35, 0.31±0.08) (*t*_[69]_ = 4.033, *p*<0.001) when tested 1 min after the last auditory pulse.
(TIF)

Figure S2 **The competitive NMDA receptor antagonist APV does not affect either the baseline response rate or rapid habituation.** (**A**) Responsiveness of zebrafish larvae following incubation with 200 µM APV (*n* = 16) or E3 (*n* = 24). The response rate of the APV group was 0.86±0.04, whereas that of the E3 control group was 0.83±0.04. These response rates were not statistically different (*t*_[38]_ = 0.49, *p*>0.5). (**B**) Following habituation training with 30 auditory pulses (1 Hz) there was no significant difference in the response rates of the APV-treated (*n* = 16, 0.25±0.11) and the E3-treated groups (*n* = 24) (0.13±0.07; *t*_[38]_ = 1.01, *p*>0.3) when tested 10 s after the last auditory pulse. (**C**) There was also no significant difference between the response rate of the APV-treated group (*n* = 16, 0.25±0.11) and the E3-treated group (*n* = 24; 0.25±0.09) (*t*_[38]_ = 0.00, *p* = 1.0) after training with 120 pulses and testing at 1 min after the last auditory pulse.
(TIF)

Methods S1 (DOCX)
