# Supplementary figures and images for: Habituation of the C-Start Response in Larval Zebrafish Exhibits Several Distinct Phases and Sensitivity to NMDA Receptor Blockade
Source: PLoS One. 2011 Dec 28;6(12):e29132. doi: 10.1371/journal.pone.0029132 (PMC3247236; doi:10.1371/journal.pone.0029132)

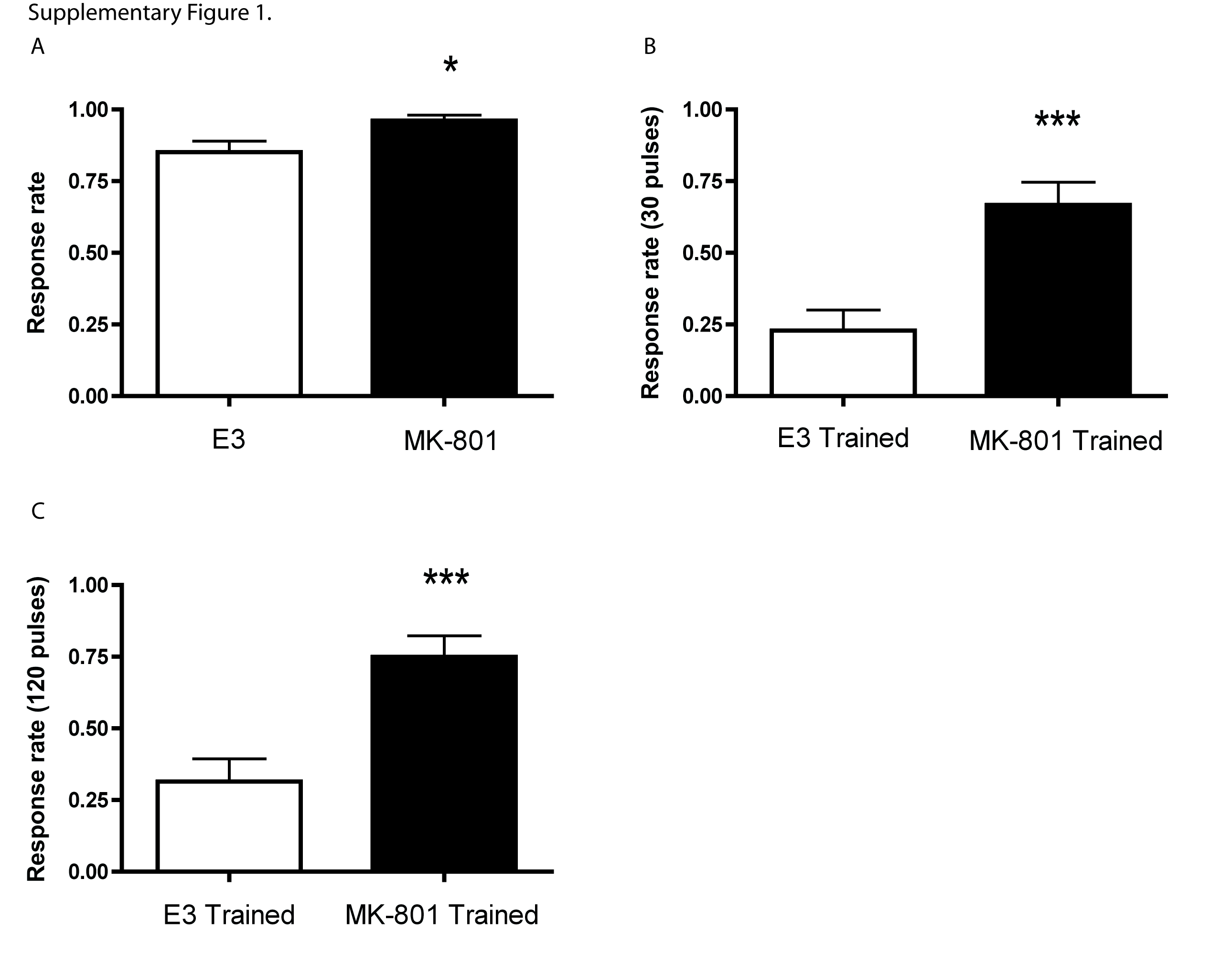

Supplement: Figure S1 — The noncompetitive NMDA receptor antagonist MK-801 enhances the baseline C-start response rate and disrupts rapid habituation. (A) Responsiveness of zebrafish larvae after incubation with 100 µM MK-801 (n = 36) or E3 (n = 35). The response rate of the MK-801 group was 0.96±0.02, whereas that of the E3 control group was 0.85±0.04 (t [69] = 2.60, p<0.05). (B) Responsiveness after habituation training with 30 pulses (1-Hz stimulation). The MK-801 group (n = 36, 0.67±0.08) was significantly more responsive than the E3 group (n = 35, 0.23±0.07) (t [69] = 4.07, p<0.001) when tested 10 s after the last auditory pulse. (C) The results following training with 120 auditory pulses (also at 1 Hz) were similar to those with the 30-stimuli protocol. Again, the MK-801 group (n = 36, 0.75±0.07) responded at a higher rate than did the E3 group (n = 35, 0.31±0.08) (t [69] = 4.033, p<0.001) when tested 1 min after the last auditory pulse. (TIF) [file pone.0029132.s001.tif]

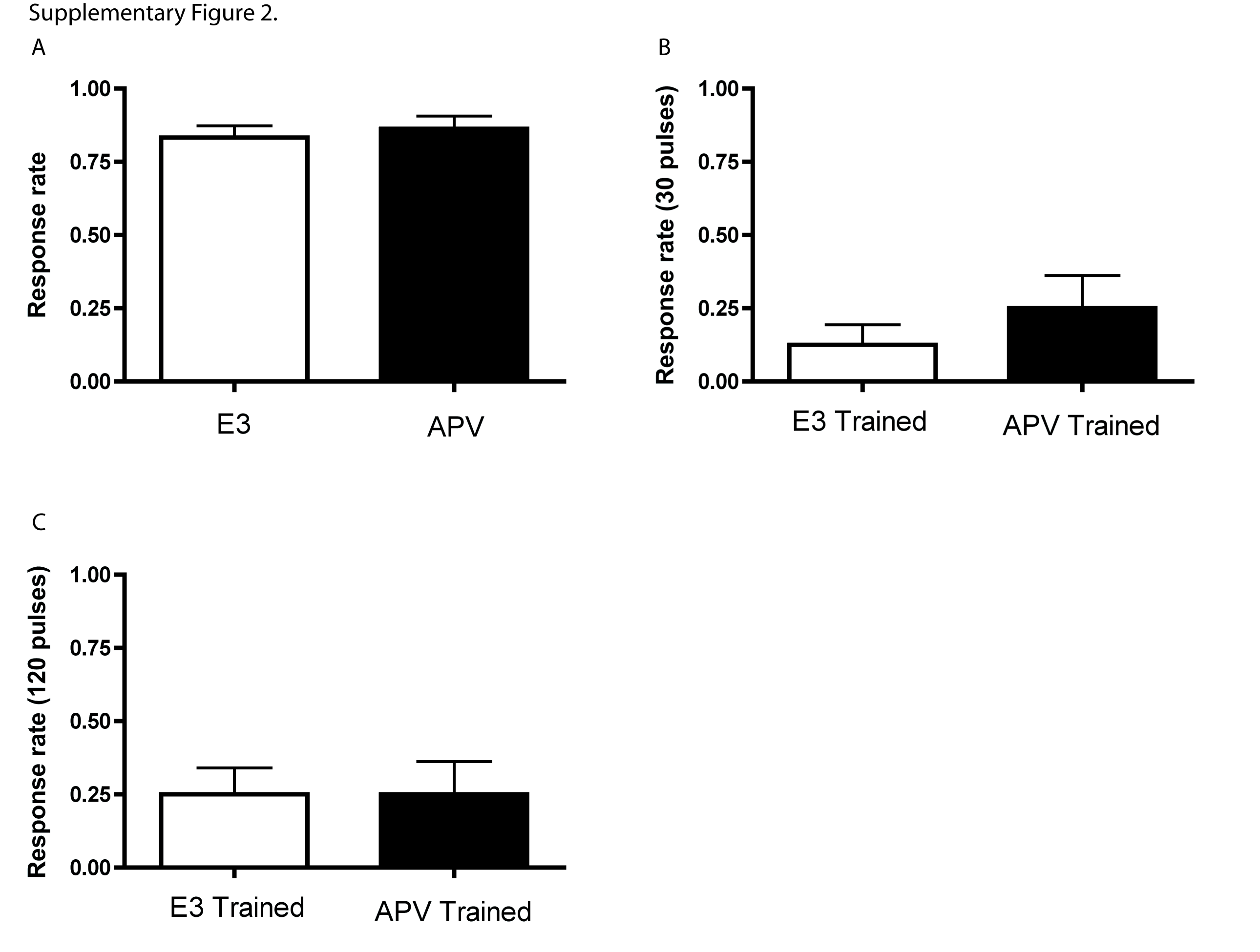

Supplement: Figure S2 — The competitive NMDA receptor antagonist APV does not affect either the baseline response rate or rapid habituation. (A) Responsiveness of zebrafish larvae following incubation with 200 µM APV (n = 16) or E3 (n = 24). The response rate of the APV group was 0.86±0.04, whereas that of the E3 control group was 0.83±0.04. These response rates were not statistically different (t [38] = 0.49, p>0.5). (B) Following habituation training with 30 auditory pulses (1 Hz) there was no significant difference in the response rates of the APV-treated (n = 16, 0.25±0.11) and the E3-treated groups (n = 24) (0.13±0.07; t [38] = 1.01, p>0.3) when tested 10 s after the last auditory pulse. (C) There was also no significant difference between the response rate of the APV-treated group (n = 16, 0.25±0.11) and the E3-treated group (n = 24; 0.25±0.09) (t [38] = 0.00, p = 1.0) after training with 120 pulses and testing at 1 min after the last auditory pulse. (TIF) [file pone.0029132.s002.tif]
