# Supplementary material for: Habituation of the C-Start Response in Larval Zebrafish Exhibits Several Distinct Phases and Sensitivity to NMDA Receptor Blockade
Source: PLoS One. 2011 Dec 28;6(12):e29132. doi: 10.1371/journal.pone.0029132 (PMC3247236; doi:10.1371/journal.pone.0029132)
Supplement: Methods S1 — (DOCX) [file pone.0029132.s003.docx]

**Supplementary Methods**

Rapid Habituation

All experimental procedures for the investigation of rapid habituation were identical to that described in the Methods section, except as indicated below. To measure the responsiveness to APV (200 µM) or MK-801 (100 µM, Sigma), we incubated larval zebrafish (6 dpf) in either the drug or control solution for 15 min prior to stimulation. APV andMK-801 were dissolved in 1% DMSO; the control solution was 1% DMSO dissolved in E3. After the end of the incubation period the baseline responsiveness of the larvae was determined by measuring the number of C-starts over 5 pretests (5 min ISI) to a standard auditory pulse (see Methods, main text). After a 15 min rest period, rapid habituation was assayed. The larvae were trained with 30 auditory pulses at 1 Hz, and their responsiveness was tested 10 s after the last training pulse. The larvae were rested for another 15 min, and then were once more assayed for rapid habituation with 120 pulses at 1 Hz followed by a posttest 1 min later.
